# Supplementary material for: Invasive and Non-Invasive Human Salmonellosis Cases Admitted between 2015 and 2021 in Four Suburban Hospitals in the Metropolitan Area of Milan (Italy): A Multi-Center Retrospective Study
Source: Pathogens. 2023 Oct 30;12(11):1298. doi: 10.3390/pathogens12111298 (PMC10674539; doi:10.3390/pathogens12111298)
Supplement: Supplementary file 1 [file pathogens-12-01298-s001.zip › pathogens-2636370-supplementary.pdf]

Supplementary Table S1. Differences in frequencies of serogroups with and without antimicrobial resistance in hospitalized patients (data available for 202 patients).

Serogroups	Antimicrobial resistance (%)	No Antimicrobial Resistance (%)	p-value
**O:4**	**78 (63,9%)**	**29 (36,3%)**	**<0,001**
**O:9**	**10 (8,2%)**	**30 (37,5%)**	**<0,001**
**O:7**	13 (10,7%)	8 (10%)	0,856
**ND**	16 (13,1%)	5 (6,3%)	0,200
**O:8**	3 (2,5%)	4 (5,0%)	0,441
**O:3,10**	0	2 (2,5%)	0,159
**O:11**	1 (0,8%)	1 (1,2%)	0,641
**O:13**	1 (0,8%)	1 (1,2%)	0,641
**O:2**	0	0	/
**Overall**	122 (100%)	80 (100%)	

Supplementary Table S2. Frequency of antibiotic resistance in O:9 isolates and other than O:9 isolates in hospitalized patients.

	Other than O:9 (n=196)	O:9 (n=56)	p-value
**AMK**	**13 (6,6%)**	**8 (14,3%)**	**0,034**
**GEN**	19 (9,7%)	9 (16,1%)	0,068
**AMC**	**51 (26,0%)**	**1 (1,8%)**	**<0,001**
**AMP**	**42 (21,4%)**	**0**	**<0,001**
**CTX**	4 (2,0%)	0	0,428
**CAZ**	6 (3,1%)	0	0,597
**CAF**	9 (4,6%)	0	0,212
**CIP**	17 (8,7%)	1 (1,8%)	0,205
**TZP**	5 (2,6%)	0	0,586
**SXT**	**18 (9,2%)**	**0**	**0,027**

AMK amikacin, AMC amoxicillin/clavulanate, AMP ampicillin, CTX cefotaxime, CAZ ceftazidime, CAF ceftriaxone, CIP ciprofloxacin, GEN gentamicin, TZP piperacillin/tazobactam, SXT trimethoprim/sulfamethoxazole
